# Supplementary material for: National Ambulance Surveillance System: A novel method using coded Australian ambulance clinical records to monitor self-harm and mental health-related morbidity
Source: PLoS One. 2020 Jul 31;15(7):e0236344. doi: 10.1371/journal.pone.0236344 (PMC7394421; doi:10.1371/journal.pone.0236344)
Supplement: Attachment — Submitted filename: PONE-D-20-00466 comments 20200120.pdf [file pone.0236344.s001.pdf]

PLOS ONE

national?

~~National Ambulance Surveillance System:~~ A novel method using (coded) Australian ambulance clinical records to monitor self-harm and mental health-related morbidity

--Manuscript Draft--

**Manuscript Number:**	PONE-D-20-00466
**Article Type:**	Research Article
**Full Title:**	National Ambulance Surveillance System: A novel method using coded Australian ambulance clinical records to monitor self-harm and mental health-related morbidity
**Short Title:**	Using ~~coded~~ National ambulance clinical records to monitor self-harm and mental health outcomes

**Corresponding Author:**	Daniel Lubman<br>Monash University<br>Richmond, VIC AUSTRALIA

**Keywords:**	suicide; self-harm; mental health; surveillance system; public health; pre-hospital ? ambulance

Abstract:

Self-harm and mental health are inter-related issues that substantially contribute to global burden of disease. However, measurement of these issues at the population level is problematic. Statistics on suicide can be captured in coronial data however, there is a significant time-lag in availability, and coronial data do not include non-fatal incidents. Although survey, emergency department, and hospitalisation data present alternative information sources to measure self-harm, these data do not include the richness of information available at the point of incident. This paper describes the mental health and self-harm modules within the National Ambulance Surveillance System (NASS), a unique Australian system for monitoring and mapping mental health and self-harm. Data are sourced from paramedic electronic patient care records provided by Australian state and territory-based ambulance services. A team of specialised research assistants use a purpose-built system to manually scrutinise and code these records. Specific details of each incident are coded, including mental health symptoms and relevant risk indicators, as well as the type, intent, and method of self-harm. NASS provides almost 90 output variables related to self-harm (i.e., type of behaviour, self-injurious intent, and method) and mental health (e.g., mental health symptoms) in the 24 hours preceding each attendance, as well as demographics, temporal and geospatial characteristics, clinical outcomes, co-occurring substance use, and self-reported medical and psychiatric history. NASS provides internationally unique data on self-harm and mental health, with direct implications for translational research, public policy, and clinical practice. This methodology could be replicated in other countries with universal ambulance service provision to inform policy and health services. ✓ Good

- coronial data
- AOD
- unique internationally
- wish there was some data! will be interesting to see how NASS data compares to "older" data
- ↑ geo spatial mapping potential discussion

Order of Authors:

Daniel Lubman

Cherie Heilbronn

Rowan P Ogeil

Jessica J Killian

Sharon Matthews

Karen Smith

Emma Bosley

Rosemary Carney

Kevin McLaughlin

Alex Wilson

Matthew Eastham

Carol Shipp

Katrina Witt

	Belinda Lloyd
	Debbie Scott

Additional Information:

Question	Response
**Financial Disclosure**<br><br>Enter a financial disclosure statement that describes the sources of funding for the work included in this submission. Review the submission guidelines for detailed requirements. View published research articles from *PLOS ONE* for specific examples.<br><br>This statement is required for submission and **will appear in the published article** if the submission is accepted. Please make sure it is accurate.<br><br>**Unfunded studies**<br>Enter: *The author(s) received no specific funding for this work.*<br><br>**Funded studies**<br>Enter a statement with the following details:<br>• Initials of the authors who received each award<br>• Grant numbers awarded to each author<br>• The full name of each funder<br>• URL of each funder website<br>• Did the sponsors or funders play any role in the study design, data collection and analysis, decision to publish, or preparation of the manuscript?<br>• **NO** - Include this sentence at the end of your statement: *The funders had no role in study design, data collection and analysis, decision to publish, or preparation of the manuscript.*<br>• **YES** - Specify the role(s) played.<br><br>* typeset	This work was supported by funding from the federal Department of Health, Movember Foundation and Beyond Blue. Funders had no influence on this manuscript or the decision to publish.
**Competing Interests**<br><br>Use the instructions below to enter a competing interest statement for this submission. On behalf of all authors, disclose any competing interests that could be perceived to bias this work—acknowledging all financial support and any other relevant financial or non-	DL has received speaking honoraria from the following: Astra Zeneca, Camurus, Indivior, Janssen-Cilag, Lundbeck, Servier and Shire, and has participated on Advisory Boards for Indivior and Lundbeck. DL and DS are investigators on an untied educational grant from Seqirus, utilising data from NASS, but is unrelated to the development of this project.

Powered by Editorial Manager® and ProduXion Manager® from Aries Systems Corporation

financial competing interests.

This statement **will appear in the published article** if the submission is accepted. Please make sure it is accurate. View published research articles from PLOS ONE for specific examples.

NO authors have competing interests

Enter: *The authors have declared that no competing interests exist.*

Authors with competing interests

Enter competing interest details beginning with this statement:

I have read the journal's policy and the authors of this manuscript have the following competing interests: [insert competing interests here]

* typeset

Ethics Statement

Enter an ethics statement for this submission. This statement is required if the study involved:

- Human participants
- Human specimens or tissue
- Vertebrate animals or cephalopods
- Vertebrate embryos or tissues
- Field research

Write "N/A" if the submission does not require an ethics statement.

General guidance is provided below. Consult the submission guidelines for detailed instructions. **Make sure that all information entered here is included in the Methods section of the manuscript.**

This project is approved through the Eastern Health Human Research Ethics Committee (HREC), with additional HREC approval for jurisdictional data provision, and requirements for informed consent were waived by these HRECs. Strict protocols are in place for data de-identification, confidentiality, storage, access and reporting. Patient identifiers are provided by some ambulance jurisdictions for the purposes of data linkage. On data receipt, these identifiers are stripped from the dataset and a unique statistical linkage key created. Identifiers are held in a password protected, secure, separate database that is accessible only to database managers. All data is de-identified prior to coding.

Powered by Editorial Manager® and ProduXion Manager® from Aries Systems Corporation

Format for specific study types

Human Subject Research (involving human participants and/or tissue)

- Give the name of the institutional review board or ethics committee that approved the study
- Include the approval number and/or a statement indicating approval of this research
- Indicate the form of consent obtained (written/oral) or the reason that consent was not obtained (e.g. the data were analyzed anonymously)

Animal Research (involving vertebrate animals, embryos or tissues)

- Provide the name of the Institutional Animal Care and Use Committee (IACUC) or other relevant ethics board that reviewed the study protocol, and indicate whether they approved this research or granted a formal waiver of ethical approval
- Include an approval number if one was obtained
- If the study involved *non-human primates*, add *additional details* about animal welfare and steps taken to ameliorate suffering
- If anesthesia, euthanasia, or any kind of animal sacrifice is part of the study, include briefly which substances and/or methods were applied

Field Research

Include the following details if this study involves the collection of plant, animal, or other materials from a natural setting:

- Field permit number
- Name of the institution or relevant body that granted permission

Data Availability

No - some restrictions will apply

Authors are required to make all data underlying the findings described fully available, without restriction, and from the time of publication. PLOS allows rare exceptions to address legal and ethical concerns. See the PLOS Data Policy and FAQ for detailed information.

Powered by Editorial Manager® and ProduXion Manager® from Aries Systems Corporation

A Data Availability Statement describing where the data can be found is required at submission. Your answers to this question constitute the Data Availability Statement and **will be published in the article**, if accepted.

Important: Stating 'data available on request from the author' is not sufficient. If your data are only available upon request, select 'No' for the first question and explain your exceptional situation in the text box.

Do the authors confirm that all data underlying the findings described in their manuscript are fully available without restriction?

Describe where the data may be found in full sentences. If you are copying our sample text, replace any instances of XXX with the appropriate details.

- If the data are **held or will be held in a public repository**, include URLs, accession numbers or DOIs. If this information will only be available after acceptance, indicate this by ticking the box below. For example: *All XXX files are available from the XXX database (accession number(s) XXX, XXX.)*.
- If the data are all contained **within the manuscript and/or Supporting Information files**, enter the following: *All relevant data are within the manuscript and its Supporting Information files.*
- If neither of these applies but you are able to provide **details of access elsewhere**, with or without limitations, please do so. For example:

  Data cannot be shared publicly because of [XXX]. Data are available from the XXX Institutional Data Access / Ethics Committee (contact via XXX) for researchers who meet the criteria for access to confidential data.

  The data underlying the results presented in the study are available from (include the name of the third party

The datasets generated and analysed for the current study are not publicly available due to the need to protect privacy and confidentiality. Ambulance data are provided to Turning Point under strict conditions for the storage, retention and use of the data. The current approval permits storage of the data at one site, Turning Point, with any analysis to be undertaken onsite, no data to be removed, and no dissemination of unit level data. Researchers wishing to undertake additional analyses of the data are invited to contact Turning Point as the data custodians.

and contact information or URL).

- This text is appropriate if the data are owned by a third party and authors do not have permission to share the data.

* typeset

Additional data availability information:

Powered by Editorial Manager® and ProduXion Manager® from Aries Systems Corporation

Manuscript

Click here to access/download;Manuscript;NAP MH SH protocol paper_20200107 noendnote.docx

National Ambulance Surveillance System: A novel method using coded Australian ambulance clinical records to monitor self-harm and mental health-related morbidity

Dan I. Lubman [1,2*], Cherie Heilbronn [1,2], Rowan P. Ogeil [1,2], Jessica J. Killian [1,2], Sharon Matthews [1,2] , Karen Smith [3,4,5], Emma Bosley [6], Rosemary Carney [7], Kevin McLaughlin [7], Alex Wilson [8], Matthew Eastham [9], Carol Shipp [10], Katrina Witt [1,2], Belinda Lloyd [1,2], Debbie Scott [1,2]

[1] Turning Point, Eastern Health, Richmond, Victoria, Australia

[2] Monash Addiction Research Centre and Eastern Health Clinical School, Monash University, Box Hill, Victoria, Australia

[3] Ambulance Victoria, Doncaster, Victoria, Australia

[4] Department of Community Emergency Health and Paramedic Practice, Monash University, Frankston, Victoria, Australia

[5] Department of Epidemiology and Preventative Medicine, Monash University, Melbourne, Victoria, Australia

[6] Queensland Ambulance Service, Brisbane, Queensland, Australia

[7] New South Wales Ambulance, Rozelle, New South Wales, Australia

[8] Ambulance Tasmania, Hobart, Tasmania, Australia

[9] St John Ambulance Australia (NT) Inc., Casuarina, Northern Territory, Australia

[10] Australian Capital Territory Ambulance Service, Fairbairn, Australian Capital Territory, Australia

Corresponding author:

*Prof Dan Lubman (DIL)

dan.lubman@monash.edu.au

Abstract

Self-harm and mental health are inter-related issues that substantially contribute to global burden of disease. However, measurement of these issues at the population level is problematic. Statistics on suicide can be captured in coronial data, however, there is a significant time-lag in availability, and coronial data do not include non-fatal incidents. Although survey, emergency department, and hospitalisation data present alternative information sources to measure self-harm, these data do not include the richness of information available at the point of incident. This paper describes the mental health and self-harm modules within the National Ambulance Surveillance System (NASS), a unique Australian system for monitoring and mapping mental health and self-harm. Data are sourced from paramedic electronic patient care records provided by Australian state and territory-based ambulance services. A team of specialised research assistants use a purpose-built system to manually scrutinise and code these records. Specific details of each incident are coded, including mental health symptoms and relevant risk indicators, as well as the type, intent, and method of self-harm. NASS provides almost 90 output variables related to self-harm (i.e., type of behaviour, self-injurious intent, and method) and mental health (e.g., mental health symptoms) in the 24 hours preceding each attendance, as well as demographics, temporal and geospatial characteristics, clinical outcomes, co-occurring substance use, and self-reported medical and psychiatric history. NASS provides internationally unique data on self-harm and mental health, with direct implications for translational research, public policy, and clinical practice. This methodology could be replicated in other countries with universal ambulance service provision to inform policy and health services.

Key words: suicide, self-harm, mental health, surveillance system, public health, ambulance

very good rationale in abstract

"Coronial" is a term specific to Australia & NZ.

+/- national "coroners' statistics" may be less confusing to a non-local audience.

Introduction

Each year suicide claims the lives of more than 800,000 people globally, with numbers increasing each year (1). In Australia, suicide is the leading cause of death for those aged from 15 to 44 years (2), costing the economy $551 million annually (3). Despite the Australian government spending an additional $47.2 million on suicide prevention in the decade to 2015-16 (4), suicide rates have not declined over that time (5). Critically, suicide deaths represent only the "tip of the iceberg". For each suicide in Australia, there are 11 hospitalisations for intentional self-harm (6) (defined as deliberate self-injury *regardless* of the degree of suicidal intent (7)), and these presentations are also increasing (8-10). In response, the World Health Organisation (WHO) has identified that monitoring morbidity-related harms as an indicator of progress towards suicide prevention is imperative (11).

Although it is possible to establish surveillance systems through the use of surveys, methodologies that capture representative populations are expensive, and must be maintained over time to enable the capture of trends and patterns. As such, most surveillance systems use routinely collected administrative data (12). For example, data on Australian suicide deaths are obtained from coronial records (13). However, difficulties in determining suicidal intent, including a lack of guidance on such deliberations (14), contribute to lag times of up to four years and renders these data unsuitable for 'real-time' suicide monitoring (15). Hospitalisation data (patients admitted for treatment) are an alternative data source, but are likely to miss a substantial number of intentional self-harm events as only those with serious physical or mental health issues are admitted for further treatment. Emergency Department (ED) data are another source, and are often used as an "early warning system" (16) to monitor intentional self-harm related presentations (17-21). ED data are more inclusive than

hospitalisation data, as patients who present to ED but are not admitted as an inpatient will be included. very true

WHO member states use the International Statistical Classification of Disease and Related Health Problems: 10th Revision (ICD-10) as an epidemiological tool to classify morbidity and mortality in health datasets. ICD-10 provides codes for injury and poisoning related harms in Chapters XIX Injury, Poisoning and Certain Other Consequences of External Causes and XX External Causes of Morbidity and Mortality (22). Although ICD was developed to monitor the prevalence of health problems in a consistent manner, for a number of reasons ICD-10 codes do not reliably capture, nor distinguish between different types of, intentional self-harm. This leads to an underestimation of self-harm in ED and hospitalisation data. First, ICD-10 codes cannot distinguish suicide attempt from self-injury without suicidal intent (23) meaning research based on ICD-10 can only report one catch-all 'intentional self-harm' variable. Further ICD-10 codes do not capture suicidal ideation at all. This is problematic as understanding the transition from suicidal ideation to suicide attempt informs effective suicide prediction and prevention activities (24). Second, an intentional self-harm injury must be clearly documented and medically treated while in ED or hospital (if admitted) in order for a code to be assigned. Therefore, if a patient either did not disclose the injury was self-inflicted or was not medically treated for the injury, then intentional self-harm ICD-10 codes may not be recorded (23). Third, some ED information systems only have capacity to record one code. Reliance on a single code means that the presence of a psychiatric disorder, suicide attempt, self-injury without suicidal intent or alcohol and other drug (AOD) intoxication, could be lost and only the physical injury that required treatment will be recorded (e.g., laceration or fracture). This is a major limitation of self-harm surveillance systems based on ICD-10 codes, and particularly for systems reliant on ED data.

Enhanced surveillance programs for self-harm, predominantly using text mining techniques to identify suicidal ideation, suicide attempt and self-injury without suicidal intent, can augment information captured in ICD-10 codes (25). However, these typically require triage or other clinical staff to complete additional training and coding. Time, resourcing, and other constraints may lead to the under-estimation of cases and there may be significant lag time for data availability (16). Natural Language Processing methodologies based on artificial intelligence (AI), offer significant improvements on text mining (26, 27). However, success of such AI methodologies are reliant on large and long-term datasets with reliably coded data to give the AI computer algorithms sufficient information to precisely replicate human coding capabilities. Also, given that only around one-third of Australians present to the ED following an episode of self-harm, there are likely many more occurrences in the community that do not result in ED presentation or hospitalisation, and are therefore not captured by either enhanced self-harm or more generalised intentional self-harm surveillance systems (28).

Routine clinical data from ambulance attendances offer a novel avenue for capturing information related to self-harm. Ambulance services are frequently the first, and sometimes the only available, healthcare service to respond to both mental health and self-harm events occurring in the community (29). Importantly, as paramedic clinical notes include observations made on scene, ambulance data provide rich information regarding the characteristics and patterns of self-harm, as well as co-occurrence with mental health symptomatology and AOD use. This paper describes the self-harm and mental health symptomatology modules of the National Ambulance Surveillance System (NASS), an established and internationally unique multi-jurisdictional surveillance system using coded ambulance clinical records.

Materials and Methods

Project development and data coverage

NASS is a surveillance system for self-harm, mental health, and AOD-related harms, created from an existing monitoring program. Project development, methodology and the AOD module of the project have been detailed previously (30-32). As the self-harm and mental health modules were added to the original AOD surveillance system (30), the system successfully leveraged existing partnerships with jurisdictional ambulance services from across Australia (ACT Ambulance Service, Ambulance Tasmania, Ambulance Victoria, NSW Ambulance, Queensland Ambulance Service, St Johns Ambulance Northern Territory, St Johns Ambulance Western Australia).

Briefly, NASS covers more than 90% of the Australian population across seven of the eight Australian states and territories (Australian Capital Territory, New South Wales, Northern Territory (from 2016), Queensland, Tasmania, Western Australia (from 2020) and Victoria). Coded NASS self-harm and mental health data are available from the pilot phase, in which the proof of concept and system feasibility were established, and an additional three financial years. From this pilot phase, 12 months of coded data is available from the state of Victoria, and data snapshots of one month per quarter (March, June, September and December) are available for other jurisdictions, except Northern Territory and Western Australia. Self-harm and mental health data for males is also available for the 2013/2014, 2015/2016 and 2016/2017 financial years, as a part of the *Beyond the Emergency* project led by Turning Point and Monash University, which investigated the scale and nature of ambulance attendances for men presenting with acute mental health issues and self-harm (33). NASS coding and reporting was approved through the Eastern Health Human Research Ethics Committee

(HREC), with data provision approved by additional HRECs as required by jurisdictional ambulance services.

Process overview

Fig 1 presents the five steps of NASS data collection and manual coding, which creates a dataset of coded ambulance attendances related to self-harm and/or mental health symptomatology. An electronic Patient Care Record (*e*PCR) was created for every ambulance attendance. Each *e*PCR included details of patient demographics, attendance location and characteristics, clinical and treatment information and outcomes and a text description of the paramedic clinical assessment. By using operational *e*PCRs that were collected for clinical purposes, no additional burden was placed on paramedics. The *e*PCRs were obtained from electronic clinical information systems used by jurisdictional ambulance services, and were provided to Turning Point, a national addiction treatment and research centre. Case coding involved manual scrutiny of each *e*PCR by specialist research assistants (RA) to establish (a) case ascertainment and (b) case classification. RAs undergo reiterative training; coding was checked for validity and the project dataset was routinely checked for anomalies. Each step, including detailed description of data filtering, processing, review and exporting, as well as RA training and review, have been previously reported (30). Coded ambulance data can be reported within three months of an ambulance attendance, depending on data provision and funder requirements.

*_*_*_**_*_*_*_**_*_*_*_**_*_*_*_**_*_*_*_**_*_*_**_*_*_*_**_*_*_*_**_*_*_*_**_*_*_*_*

Insert Fig 1. National Ambulance Surveillance System data collection and coding process

*_*_*_**_*_*_*_**_*_*_*_**_*_*_*_**_*_*_*_**_*_*_**_*_*_*_**_*_*_*_**_*_*_*_**_*_*_*_*

Case ascertainment

Inclusion criteria were met if self-harm or mental health symptomatology contributed to the ambulance attendance, using the core inclusion criterion: 'Is it reasonable to attribute a recent (past 24-hours) incident of self-harm or symptom of mental health as contributing to the ambulance attendance?' This information was ascertained through manual scrutiny of each *e*PCR, considering paramedic clinical assessment, patient self-report, information from third parties and other evidence at the scene, such as written statements of intent (including social media, text messages and written notes), as recorded by paramedics in the *e*PCR. This evidence was also used to determine method of self-harm as well as presence of risk indicators.

Case classification

Self-harm related ambulance attendances

Self-harm related ambulance attendances were classified by the presence of self-harm preceding (past 24 hours) or during the ambulance attendance, with four categories of self-harm related ambulance attendances defined and coded as: (a) self-injury (known as non-suicidal self-injury in the USA): non-fatal intentional injury without suicidal intent (34); (b) suicidal ideation: thinking about killing oneself without acting on the thoughts (35); (c) suicide attempt: non-fatal intentional injury with suicidal intent, regardless of likelihood of lethality (35); (d) suicide: fatal intentional injury with suicidal intent (35). Suicide, suicide attempt and suicidal ideation were mutually exclusive, however, self-injury could be simultaneously coded with any other self-harm case category.

Self-harm method and suicidal ideation preparation

Common methods of self-harm were modified from the ICD-10 External Cause Codes (22) and multiple methods could be coded within one case. Thirteen methods of suicide, suicide attempt or suicidal ideation were defined and coded as: (a) intentional AOD poisoning (purposeful AOD consumption with suicidal intent); (b) carbon monoxide (CO) poisoning; (c) other poisoning (excluding intentional AOD poisoning and CO poisoning); (d) hanging; (e) asphyxia (excluding hanging); (f) laceration or penetrating wound; (g) firearm discharge; (h) drowning; (i) jumping from a height; (j) vehicular impact; (k) burn or corrosions; (l) other method; (m) unknown method. Seven methods of self-injury are defined and coded as: (a) laceration or penetrating wound; (b) bodily impact; (c) burn or corrosions; (d); ingestion of foreign object/s; (e) intentional AOD poisoning; (f) other method; (g) unknown method. Three categories of suicidal ideation preparation were defined and coded: (a) planned; (b) unplanned; (c) unknown if planned.

AOD poisoning: overdose threshold met

To compliment the AOD module of the NASS (30), a supplementary category classifying the collective impact of substance use in AOD poisoning was defined and coded: AOD poisoning (overdose threshold met). This 'overdose threshold met' coding category applies to intentional AOD poisoning (defined in this paper), as well as two coding categories described in a previous paper ((a) unintentional AOD poisoning: purposeful AOD consumption without suicidal intent; (b) undetermined intent AOD poisoning: purposeful AOD consumption with unknown suicidal intent (where determination of intentional or unintentional AOD poisoning cannot be made from the *e*PCR)) (23). AOD poisoning (overdose threshold met) case inclusion criteria varies depending on the type of drug consumed, using proxy measures to identify cases with potential for medical harm. For alcohol and illicit substances, a potentially life-threatening event was identified by a clinical picture involving a Glasgow Coma Scale (GCS)

score of less than nine (36), low respiratory rate and/or paramedic concern for securing an airway. For pharmaceutical preparations, a concordant clinical picture to alcohol or illicit drug overdose, or the consumption of 10 or more times the typically prescribed dose was used to determine AOD poisoning. Case inclusion criteria for pharmaceutical drugs varies from that of alcohol and illicit substances due to the complexity of considering total drug effect for individual pharmaceutical preparations during the manual coding process (30).

Mental health-related ambulance attendances

Mental health-related ambulance attendances were classified by the presence of a mental health symptom preceding or during the ambulance attendance and, importantly, did not equate to a diagnosis. Four categories of mental health-related ambulance attendances were defined and categorised as: (a) anxiety: overwhelming and intrusive worry, and/or panic attack symptom profile; (b) depression: symptom profile consistent with depression, such as low mood, feelings of hopelessness, despair, worthlessness, anhedonia, change in sleep and/or appetite; (c) psychosis: presence of hallucinations or delusions; (d) other mental health symptom: mental health symptoms not otherwise unspecified. Importantly, cases where presenting mental health symptoms were likely to have a medical cause (e.g., hypoxia, head injury, delirium, diabetes and dementia), rather than a mental health cause, were excluded.

Mental health and other risk indicators

For cases ascertained to meet inclusion criteria for mental health-, self-harm- or AOD-related ambulance attendances, 41 risk indicators are also coded as 'recorded' or 'not recorded'. Risk indictors were defined and coded into four broad categories, with individual risk factors presented in Table 1: (a) history of self-harm; (b) history of mental health symptoms or

diagnosis; (c) concurrent risk indictor: experiencing the risk indicator at the time of ambulance attendance (d) lifetime risk indictor: have experienced the risk indictor during their lifetime.

Output variables

The self-harm and mental health modules of NASS capture more than 80 output variables, in addition to the demographic and scene information that was consistent across all modules, including patient details, scene details, and the physical condition of the patient. The self-harm module had 35 variables that categorise the type of self-harm, intent, and method. The mental health module had 46 variables; five that described mental health symptoms at the time of the ambulance attendance, and 31 that described risk indicators. Output variables related to these modules are summarised in Table 1.

Table 1. Self-harm and mental health variables available in the NASS dataset

**Patient and case details**			
**Case details**	**Patient details**	**Scene details**	**Physical condition**
Case number	Gender	*Public / private*	Fatal event
Case date and time	Age	*Indoor / outdoor*	*Pulse rate*
Transport to hospital	*Residential postcode*	*Event postcode and coordinates*	*Respiratory rate*
Non-transport reason		Police co-attendance	*GCS*
		Others on scene	*Naloxone administered*
		Minors on scene	Naloxone responsive
**Case classification during ambulance attendance**			
**Mental health**	**Self-harm**		
	**Self-harm and other AOD poisoning**	**Suicidal intent or planning***	**Self-harm method****
Anxiety	Suicide	Not applicable	Intentional AOD poisoning<br>Hanging<br>Vehicular impact<br>Laceration/penetrating wound<br>Jumping from height<br>CO poisoning<br>Other poisoning<br>Firearm<br>Drowning<br>Burning<br>Asphyxia<br>Other
Depression	Suicide attempt	Evidence of intent<br>Evidence of intent, but denied	
Psychosis	Suicidal ideation	Suicide plan<br>No suicide plan<br>Unknown if plan exists	

Other/unspecified	Self-injury	Evidence of intent<br>Evidence of intent, but denied	Intentional AOD poisoning<br>Laceration/penetrating wound<br>Burning<br>Asphyxia<br>Bodily impact<br>Ingestion of foreign body#<br>Other
	Unintentional AOD poisoning	n/a	
	Undetermined intent AOD poisoning	n/a	
**Relevant history and risk indicators**			
**Self-harm history**	**Mental health history**	**Current risk indicators**	**Lifetime risk indicators**
Suicide attempt	Anxiety	Agitation	Culturally/linguistically diverse
Suicidal ideation	Post-traumatic stressor disorder	Poor social support	Military service history
Self-injury	Obsessive compulsive disorder	Emergency MH team	Foster care/state guardianship
AOD poisoning: unintentional/ undetermined intent	Bipolar disorder	Link to health services	Post prison release
	Depression	Housing problem	Refugee background
	Schizophrenia	Unemployment	Suicidal exposure
	Other/unspecified psychosis	Bereavement	Intellectual impairment
	Borderline personality disorder	Family problem	Acquired brain injury
	Other personality disorder	Chronic pain	Dementia
	AOD misuse	Sleeping problems	Developmental disorder
	Eating disorder	Financial problems	
	Other / unspecified indicator	Gambling problems	
		In custody	
		Bullying	
		Other / unspecified indicator	

* Intent relates to suicidal attempt and self-injury; planning relates to suicidal ideation

**For suicide, suicide attempt and suicidal ideation, the method pertains to the self-harm method that was undertaken by the patient. For suicidal ideation, the method pertains to the self-harm method that was planned by the patient

#Excludes AOD or other poisons

Variables that are used directly from ambulance service data provision, and do not undergo additional coding within the NASS, are shown in italics

Ethics approval

This project is approved through the Eastern Health Human Research Ethics Committee (HREC), with additional HREC approval for jurisdictional data provision, and requirements for informed consent were waived by these HRECs. Strict protocols are in place for data de-identification, confidentiality, storage, access and reporting. Patient identifiers are provided by some ambulance jurisdictions for the purposes of data linkage. On data receipt, these identifiers are stripped from the dataset and a unique statistical linkage key created. Identifiers are held in a password protected, secure, separate database that is accessible only to database managers. All data is de-identified prior to coding.

Results and Discussion

NASS provides unique and timely monitoring of acute self-harm and mental health morbidity, and covers more than 90% of Australia's population. NASS also captures a greater proportion of the community experiencing self-harm and mental ill health than surveillance systems that use ED, hospital admission or coronial data. These highly relevant and valuable data are not captured by other means, and strengthen our understanding of the context and burden of self-harm and mental health conditions on individuals, the community, health services, and particularly ambulance responses to acute crises. Further, as mental health symptomatology and suicidal intent fluctuate in both duration and intensity, paramedics' ability to assess these outcomes as close in time as possible to the time of the event are a valuable context for improved public health policy recommendations.

NASS data have already underpinned and evaluated a major mental health and wellbeing initiative, *Beyond the Emergency*, a program improving the well-being of men by linking

ambulance patients to low-cost mental health interventions and providing mental health training to Australian paramedics (33). Furthermore, these data have monitored spatial and temporal trends in self-harm and mental health-related harms (37), and underpinned a range of research to guide public policy, such as self-harm and mental health related harms in pre-adolescents (38), self-harm and mental health related harms that co-occurs with inhalant misuse (39), the prevalence of a history of self-harm and/or mental health in attendances relating to pregabalin misuse (40), the co-occurrence of psychosis symptoms in ambulance attendances related to methamphetamine use (41), and the role of sleep and co-morbid mental health and AOD-factors in suicide ideation and attempt related ambulance attendances (42).

System aptitude to delineate types of self-harm and mental health symptomatology, along with method of self-harm and risk indicators, sets it apart from other population level data sources. For example, Fig 2 shows self-harm related ambulance attendances from the three largest jurisdictions (New South Wales, Queensland and Victoria). This highlights NASS's capacity to identify types of behaviours across the spectrum of self-harm, including self-injury, suicidal ideation and suicide attempt. Demarcation of self-harm type facilitates targeted intervention points and strategies, enhances evaluation of prevention programs, and provides much needed evidence to further clarify the contentious issue of predictive value of previous self-harm on subsequent suicide attempts and fatal suicide (43). There is a noticeable difference in the rates of specific types of self-harm across the jurisdictions, such as higher rates in Queensland. There are many contributing factors to this, such as treatment access and provision across jurisdictions (e.g., lower access to 24-hour health centres in states such as Queensland that have a higher regional and remote population, and greater geographic spread).

*_*_*_**_*_*_*_**_*_*_*_**_*_*_*_**_*_*_*_**_*_*_**_*_*_*_**_*_*_*_**_*_*_*_**_*_*_*_*

Insert Fig 2. Types of self-harm related ambulance attendances per 100,000 population in Victoria, Queensland and New South Wales, 2016 snapshot months (Mar, Jun, Sep, Dec)

*_*_*_**_*_*_*_**_*_*_*_**_*_*_*_**_*_*_*_**_*_*_**_*_*_*_**_*_*_*_**_*_*_*_**_*_*_*_*

Fig 3 shows the proportion by which specific types of self-harm have co-occurring current mental health symptoms and/or AOD use, previous suicide attempts and, for suicidal ideation, whether a current suicide plan is in place. This figure supports assertions from other sources that a high proportion of those who die by suicide in high income countries have a mental health diagnoses (44) or substance use disorders (45). Importantly, our data identifies patterns of co-morbidity during an acute crisis to inform interventions, such as the low-cost universal intervention Turning Point and Monash University trialled in *Beyond the Emergency*, to improve male mental health and well-being (33). Conversely, as these data can similarly be used to explore self-harm events without co-occurring or historical mental health symptoms, there are also positive implications for research into impulsivity and self-harm.

*_*_*_**_*_*_*_**_*_*_*_**_*_*_*_**_*_*_*_**_*_*_**_*_*_*_**_*_*_*_**_*_*_*_**_*_*_*_*

Insert Fig 3. Self-harm types by current mental health and AOD comorbidity and historical suicide attempts in 2016/2017 financial year

*_*_*_**_*_*_*_**_*_*_*_**_*_*_*_**_*_*_*_**_*_*_**_*_*_*_**_*_*_*_**_*_*_*_**_*_*_*_*

The type of substances most frequently co-occurring with suicidal ideation and suicide attempt-related ambulance attendances are shown in Fig 4a, and Fig 4b shows data utility by drilling down to individual analgesic medications that were consumed during a self-harm event. These data augment understanding of AOD poisoning as an intentional self-harm method, as well as allowing investigation of substance use associated with and preceding self-harm. Because specific pharmaceutical preparations are coded (described elsewhere (30)), rather than broad pharmaceutical groupings, it is possible to make explicit recommendations for pharmaceutical regulatory schemes and prescribing guidelines to reduce self-harm. For

example, these data provided evidence to inform inclusion of specific pharmaceutical medications during the development of Victoria's real-time prescription monitoring program (46). Capacity to examine acute AOD use (including alcohol, specific illicit drugs and individual pharmaceutical medications) immediately prior to self-harm is significant. Research has historically focused on the well-recognised interactions of AOD dependence and self-harm, but the relationship between acute ingestion and self-harm is patchy. Consequently, AOD use unrelated to use disorders can be overlooked as a clinically relevant issue once the acute intoxication has resolved (47). Yet, acute alcohol use prior to a suicide attempt has recently been estimated to increase the risk of suicide attempt by seven, with an increase to 37 times the risk if alcohol consumption is heavy (48). Emerging evidence has also shown that acute use of other central nervous system (CNS) depressants, including illicit and pharmaceutical opioids and sedatives/anxiolytics, have almost three times the risk of suicide attempt (49) compared to those who have not used CNS depressants. Our data provides new avenues to examine these associations. good

*_*_*_**_*_*_*_**_*_*_*_**_*_*_*_**_*_*_*_**_*_*_**_*_*_*_**_*_*_*_**_*_*_*_**_*_*_*

Insert Fig 4. Fig 4(a). Drug categories involved in Victorian suicidal ideation and suicide attempt-related ambulance attendance, 2012 to 2018; Fig 4(b). Individual analgesic drugs involved in Victorian suicide attempt-related ambulance attendances, 2012 to 2018

*_*_*_**_*_*_*_**_*_*_*_**_*_*_*_**_*_*_*_**_*_*_**_*_*_*_**_*_*_*_**_*_*_*_**_*_*_*

The innovative geo-spatial mapping capability off this surveillance system was enabled by uptake of geo-coordinate collection by some Australian ambulance services. When combined with the other coded variables in these data, these coordinates allow for identification of local environmental risk factors that are associated with self-harm, for example bridges or rail lines, so that environmental methods can be employed to reduce suicide clusters.

? pretty important... may deserve more commentary for its public health/privacy implications

Limitations and Future Directions

Data are collected for operational rather than research purposes with paramedics only recording information that they observe, or is provided to them by the patient or bystanders, and which they deem clinically relevant to patient care. It is possible that relevant information with respect to self-harm or mental health variables is not recorded, or similar events may not be recorded consistently by different paramedics over time. Close partnerships with jurisdictional ambulance services ensures ongoing, and changing, paramedic and data warehouse operations are clearly understood by the research team. Intra- and inter-rater reliability of the data is maintained by comprehensive training and reliability audits, as outlined in previous publications (30).

NASS data only includes those cases serious enough to require ambulance attendance. Inherently, this dataset is primarily a morbidity dataset as fatal suicide is under-represented as ambulances do not attend all deaths, and when they do attend there may be insufficient information to determine suicidal intent at the scene. However, paramedic clinical records are rich sources of information that complement existing population health metrics (e.g., hospital and ED presentations). Analysis of these records allow for the identification of numerous risk factors and associated drivers of suicide and self-harm that have potential for intervention either as preventive strategies or treatment options. Importantly, the data are collected in a manner that is not intrusive or demanding on those affected by suicide and self-harm, and does not rely on additional data collection by, or interactions with, already burdened health services.

Transformation of these data into a publicly available, online surveillance resource could be modelled on the ambulance component of *AODstats.org.au*, the online dissemination platform for the research team's previous AOD surveillance program (30). This would enhance

self-harm and mental health policy formulation and evaluation at a local, state and national level. The timely nature of the system means data could be available to stakeholders within three to six months of an ambulance attendance, with data uploaded online shortly thereafter. This is significantly more timely than other surveillance systems, including those using coronial data and enhanced self-harm surveillance systems, with significant time lags (15) (16).

Conclusions

NASS data provides a population based, cost-efficient resource that can be used to inform the development of prevention initiatives, and also serve to evaluate policies and practice over time, specific geographic regions and population groups. NASS is dynamic and changes can be made in response to emerging or changing mental health harms or priorities. In order to improve the utility of NASS, future work will focus on data linkage and the use of artificial intelligence to assist in screening and coding the data, thereby increasing the timeliness and completeness of the data.

Acknowledgements

We acknowledge the RA team in Population Health at Turning Point, Mr Mark Hoffman the database manager, and our partner ambulance services and paramedics. We also acknowledge Isabelle Hum, who created Figure 3.

References

1. Naghavi M. Global, regional, and national burden of suicide mortality 1990 to 2016: Systematic analysis for the Global Burden of Disease Study 2016. BMJ. 2019;364:194.

2. Australian Bureau of Statistics. 3303.0 - causes of death, Australia, 2017. Canberra, ACT: Australian Bureau of Statistics; 2017.

3. Kinchin I, Doran C. The cost of youth suicide in Australia. Int J Environ Res Pub Health. 2018;15:672.

4. Australian Institute of Health and Welfare (AIHW). Australia's Health 2018. Canberra, ACT: AIHW; 2018.

5. Snowdon J. Should the recently reported increase in Australian suicide rates alarm us? Aust NZ J Psychiatry. 2016;51:766-9.

6. Harrison J, Henley G. Suicide and Hospitalised Self-Harm in Australia: Trends and Analysis. Canberra, ACT: Australian Institute of Health and Welfare; 2014.

7. Hawton K, Zahl D, Weatherall R. Suicide following deliberate self-harm: long-term follow-up of patients who presented to a general hospital. Br J Psychiat. 2003;182:537-42.

8. Kolves K, Crompton D, Turner K, Stapelberg NJ, Khan A, Robinson G, et al. Trends and repetition of non-fatal suicidal behaviour: analyses of the Gold Coast University Hospital's Emergency Department. Aust Psychiat. 2018;26(2):170-5.

9. Leckning BA, Li SQ, Cunningham T, Guthridge S, Robinson G, Nagel T, et al. Trends in hospital admissions involving suicidal behaviour in the Northern Territory, 2001-2013. Aust Psychiat. 2016;24(3):300-4.

10. Perera J, Wand T, Bein KJ, Chalkley D, Ivers R, Steinbeck KS, et al. Presentations to NSW emergency departments with self-harm, suicidal ideation, or intentional poisoning, 2010-2014. Med J Aust. 2018;208:348-53.

11. World Health Organisation. Preventing suicide: A global imperative. Geneva: World Health Organisation; 2014.

12. Scott D, Faulkner A. The role, importance and challenges of data for a public health model. In: Higgins D, Lonne B, Herronkohl T, Scott D. (Eds). Revisioning a public health approach to child protection. Denver, Colorado: Springer; 2019.

13. De Leo D, Dudley M, Aebersold C, Mendoza J, Barnes M, Harrison J, et al. Achieving standardised reporting of suicide in Australia: rationale and program for change. Med J Aust. 2010;192:452-6.

14. Jowett S, Carpenter B, Tait G. Determining a suicide under Australian law. Uni NSW Law J. 2018;41:1-25.

15. Studdert D, Walter S, Kemp C, Sutherland G. Duration of death investigations that proceed to inquest in Australia. Inj Prevent. 2016;22:314-20.

16. Witt K, Robinson J. Sentinel surveillance for self-harm: Existing challenges and opportunities for the future. Crisis. 2019;40:1-6.

17. Whyte I, Dawson A, Buckley N, Carter G, Levey C. A model of care for the management of self-poisoning. Med J Aust. 1997;167:142-6.

18. Williams S. Establishing a self-harm surveillance register to improve care in a general hospital. Br J Ment Health Nurs. 2015;4:20-5.

19. Perry I, Corcoran P, Fitzgerald A, Keeley H, Reulbach U, Arensman E. The incidence and repetition of hospital-treated self-harm: Findings from the world's first national registry. PLoS One. 2012;7:e31663.

20. Hawton KB, H, Casey D, Simkin S, Palmer B, Cooper J, Kapur N, et al. Self-harm in England: a tale of three cities. Soc Psychiat Epidemiol. 2007;42:513-21.

21. Metzger MH, Tvardik N, Gicquel Q, Bouvry C, Poulet E, Potinet-Pagliaroli V. Use of emergency department electronic medical records for automated epidemiological surveillance of suicide attempts: a French pilot study. Int J Meth Psychiat Res 2017;26(2).

22. National Casemix and Classification Centre (NCCC). The international statistical classification of diseases and related health problems, 8th revision, Australian modification (ICD-10-AM). Wollongong: NCCC, The University of Wollongong; 2013.

23. Hedegaard H, Schoenbaum M, Claassen C, Crosby A, Holland K, Proescholdbell S. Issues in developing a surveillance case definition for nonfatal suicide attempt and intentional self-harm using International Classification of Diseases, Tenth Revision, Clinical Modification (ICD-10-CM) coded data. Natl Health Stat Report. 2018;108:1-19.

24. Klonsky E, Saffer B, Bryan C. Ideation-to-action theories of suicide: a conceptual and empirical update. Curr Opin Psychol. 2017;22:38-43.

25. Watson W, Ozanne-Smith J. Injury surveillance in Victoria, Australia: developing comprehensive injury incidence estimates. Accident Anal Prev. 2000;32:277-86.

26. McKenzie K, Campbell MA, Scott DA, Discoll TR, Harrison JE, McClure RJ. Identifying work related injuries: comparison of methods for interrogating text fields. BMC Med Inform Decis Mak. 2010;10:19.

27. Faverjon C, Berezowski J. Choosing the best algorithm for event detection based on the intended application: A conceptual framework for syndromic surveillance. J Biomed Informat. 2018;85:126-35.

28. De Leo D, Cerin E, Spathonis K, Burgis S. Lifetime risk of suicidal ideation and attempts in an Australian community: Prevalence, suicidal process, and help-seeking behaviour. J Affect Disord. 2005;86:215-24.

29. Roggenkamp R, Andrew E, Nehme Z, Cox S, Smith K. Descriptive analysis of mental health-related presentations to emergency medical services. Prehosp Emerg Care. 2018;22(4):399-405.

30. Lubman DI, Matthews S, Scott D, Heilbronn C, Killian JJ, Ogeil RP, Witt K, Crossin R, Smith K, Bosley E, Carney R, Wilson A, Eastham M, Keene T, Brook, C, Lloyd, B. The National Ambo Project: a novel surveillance system for monitoring acute harms related to alcohol, illicit and pharmaceutical drug consumption using coded Australian ambulance clinical records. PloS One. Revised 17/12/19.

31. Dietze P, Jolley D, Cvetkovski S, Cantwell K, Jacobs I, Indig D. Characteristics of non-fatal opioid overdoses attended by ambulance services in Australia. Aust NZ J Pub Health. 2004;28:569-75.

32. Lloyd B, Matthews S, Gao CX, Heilbronn C, Beck D. Trends in alcohol and drug related ambulance attendances in Victoria: 2013/14. Fitzroy, Victoria: Turning Point; 2015.

33. Turning Point. Beyond the Emergency: a national study of ambulance responses to men's mental health. Richmond, Victoria, Australia; 2019.

34. Nock MK, Favazza AR. Nonsuicidal self-injury: definition and classification. In: Nock MK. (Ed.). Understanding non-suicidal self-injury: origins, assessment, and treatment. Washington (DC): American Psychological Association; 2009. p. 9–18.

35. Klonsky ED, May AM, Saffer BY. Suicide, Suicide Attempts, and Suicidal Ideation. Ann Rev Clin Psychol. 2016;12:307-30.

36. Teasdale G, Murray G, Parker L, Jennett B. Adding up the Glasgow Coma Score. Acta Neuro Suppl. 1979;28(1):13-6.

37. Lloyd B, Gao CX; Heilbronn C, Lubman DI. Self-harm and mental health-related ambulance attendances in Australia - 2013 data. Fitzroy, Victoria: Turning Point; 2015.

38. Scott D, Crossin R, Ogeil R, Smith K, Lubman D. Exploring harms experienced by children aged 7 to 11 using ambulance attendance data: a 6-Year comparison with adolescents aged 12–17. Int J Environ Res Publ Health. 2018;15:1385.

39. Crossin R, Scott D, Witt KG, Duncan JR, Smith K, Lubman DI. Acute harms associated with inhalant misuse: co-morbidities and trends relative to age and gender among ambulance attendees. Drug Alcohol Depend. 2018;190:46-53.

40. Crossin R, Scott D, Arunogiri S, Smith K, Dietze PM, Lubman DI. Pregabalin misuse-related ambulance attendances in Victoria, 2012–2017: characteristics of patients and attendances. Med J Aust. 2019; 210:75-79.

41. Arunogiri S, Gao CX, Lloyd B, Smith K, Lubman DI. The role of methamphetamines in psychosis-related ambulance presentations. Aust NZ J Psychiat. 2015;49:939-40.

42. Ogeil RP, Witt K, Scott D, Smith K, Lubman DI. Self-reported Sleep disturbance in ambulance attendances for suicidal ideation and attempted suicide between 2012 and 2017. J Affect Dis. 2019. https://doi.org/10.1016/j.jad.2019.11.158

43. Ribeiro JD, Franklin JC, Fox KR, Bentley KH, Kleiman EM, Chang BP, et al. Self-injurious thoughts and behaviors as risk factors for future suicide ideation, attempts, and death: a meta-analysis of longitudinal studies. Psychol Med. 2016;46(2):225-36.

44. Cavanagh JT, Carson AJ, Sharpe M, Lawrie SM. Psychological autopsy studies of suicide: a systematic review. Psychol Med. 2003;33(3):395-405.

45. Ferrari A, Norman R, Freedman G, Baxter A, Pirkis J, Harris M, et al. The burden attributable to mental and substance use disorders as risk factors for suicide: findings from the global burden of disease study 2010. PLoS One. 2014;9:e91936.

46. Liew D, Joules E, Booth J, Garrett K, Frauman A. Evidence to inform the inclusion of schedule 4 prescription medications on a real-time prescription monitoring system Melbourne: Austin Health; 2017.

47. Kalk NJ, Kelleher MJ, Curtis V, Morley KI. Addressing substance misuse: a missed opportunity in suicide prevention. Addiction. 2019;114(3):387-8.

48. Borges G, Bagge CL, Cherpitel CJ, Conner KR, Orozco R, Rossow I. A meta-analysis of acute use of alcohol and the risk of suicide attempt. Psychol Med. 2017;47(5):949-57.

49. Bagge CL, Borges G. Acute Substance Use as a Warning Sign for Suicide Attempts: A Case-Crossover Examination of the 48 Hours Prior to a Recent Suicide Attempt. J Clin Psychiat. 2017;78(6):691-6.

Figure 1

Click here to access/download;Figure;Lubman_Figure 1.png

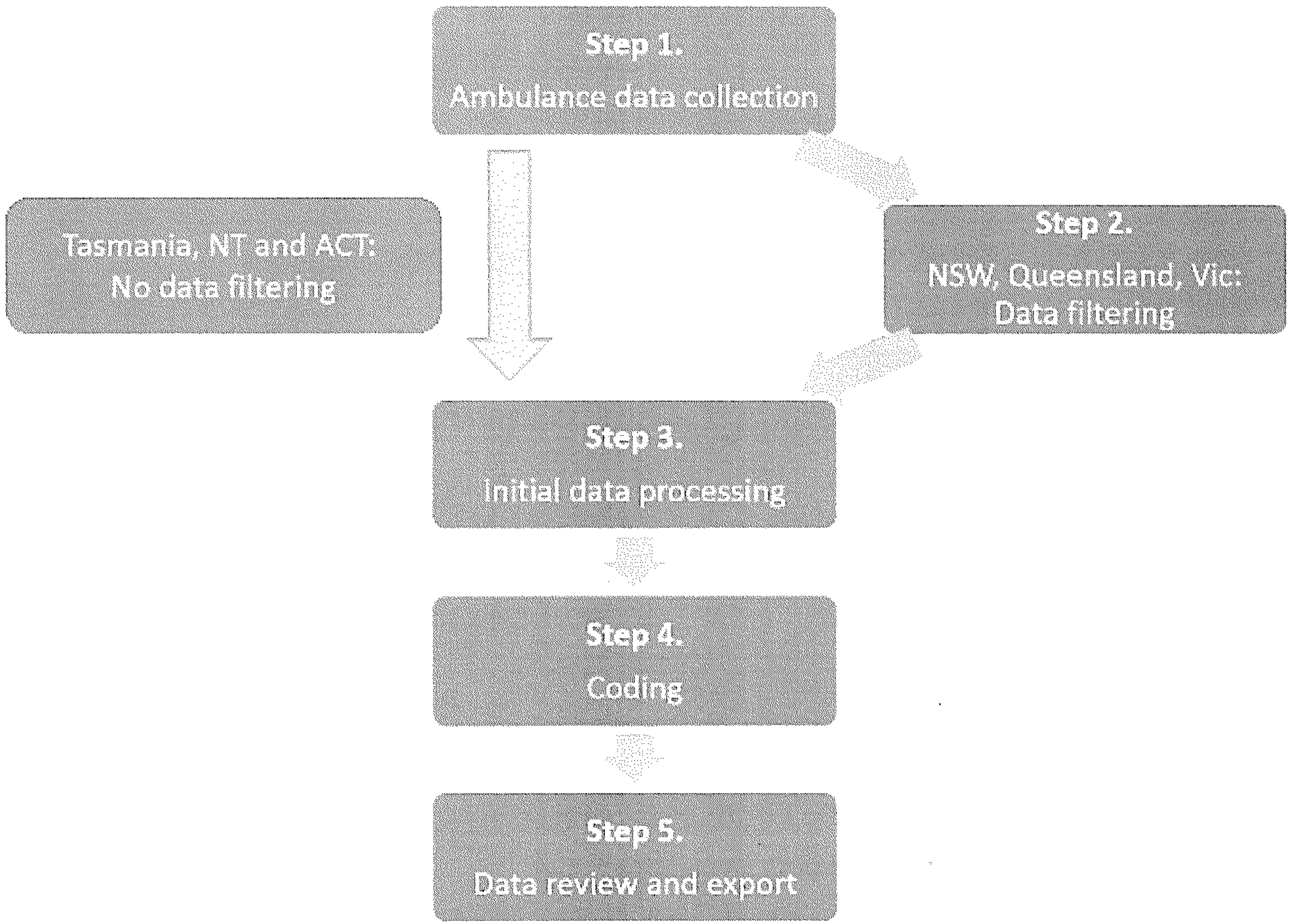

Figure 2

Click here to access/download;Figure;Lubman_Figure 2.tif

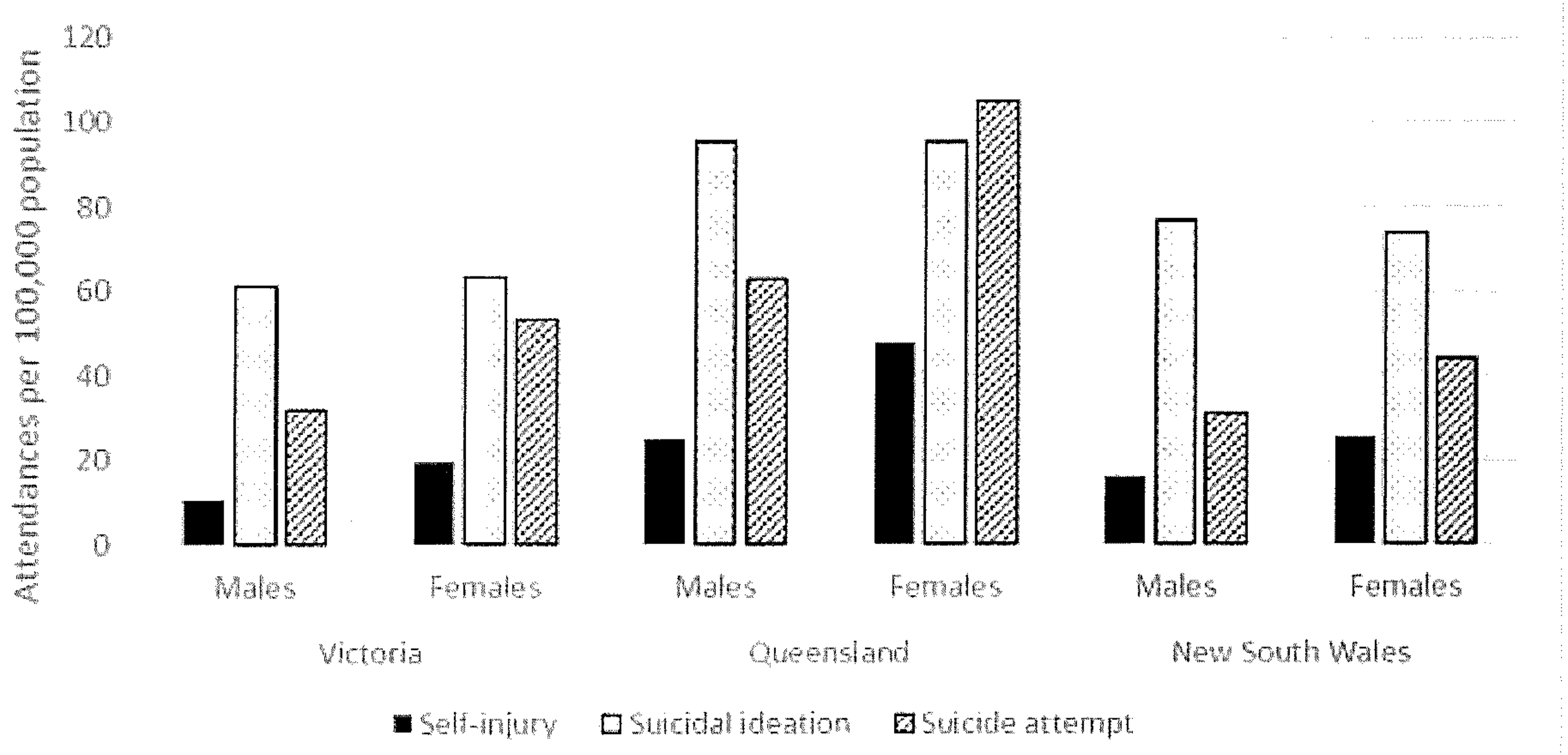

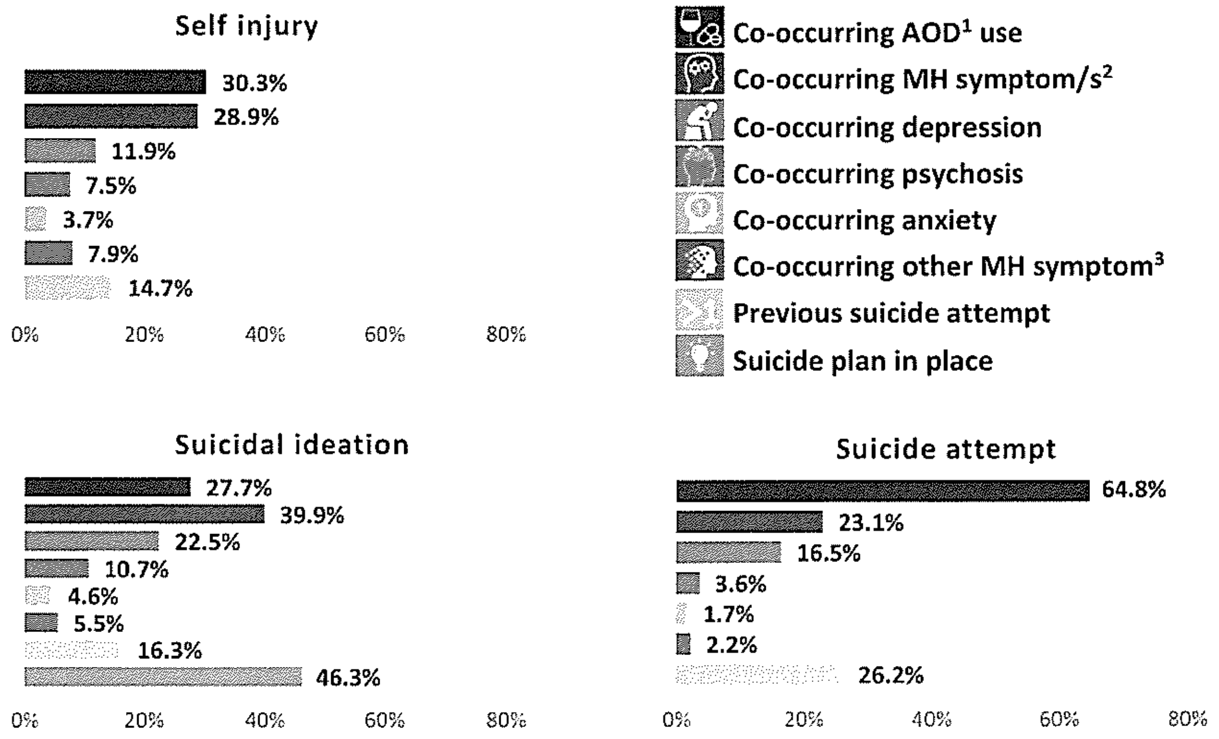

[1] Alcohol and other drugs
[2] Any current mental health symptom
[3] Other current mental health symptom/s, excluding anxiety, depression and/or psychosis

Figure 4

Click here to access/download;Figure;Lubman_Figure4.tif

Figure 4 (a) Drug categories involved in Victorian suicidal ideation and suicide attempt-related ambulance attendance, 2012 to 2018

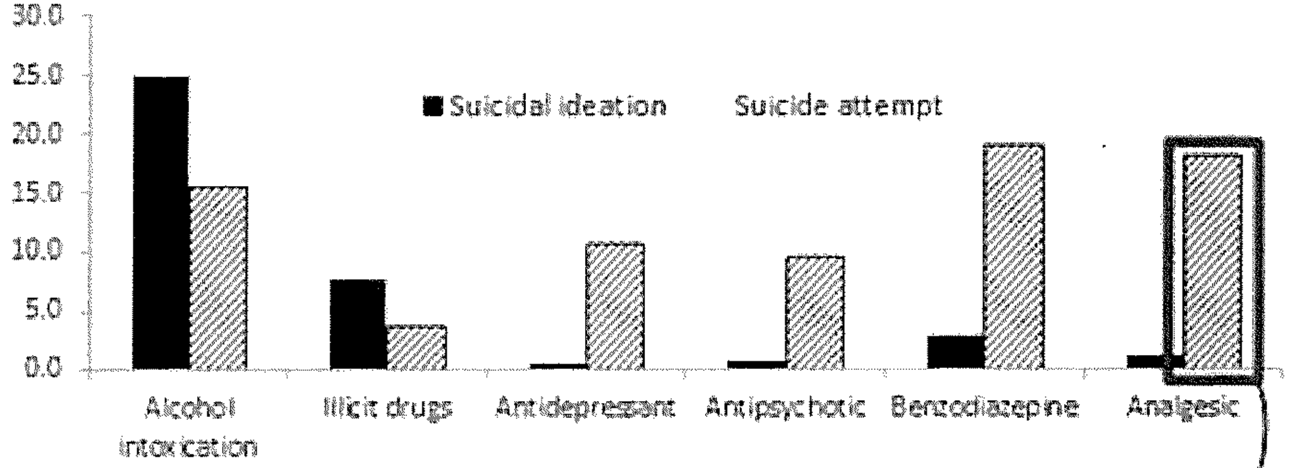

Figure 4 (b) Individual analgesic drugs involved in Victorian suicide attempt-related ambulance attendances, 2012 to 2018

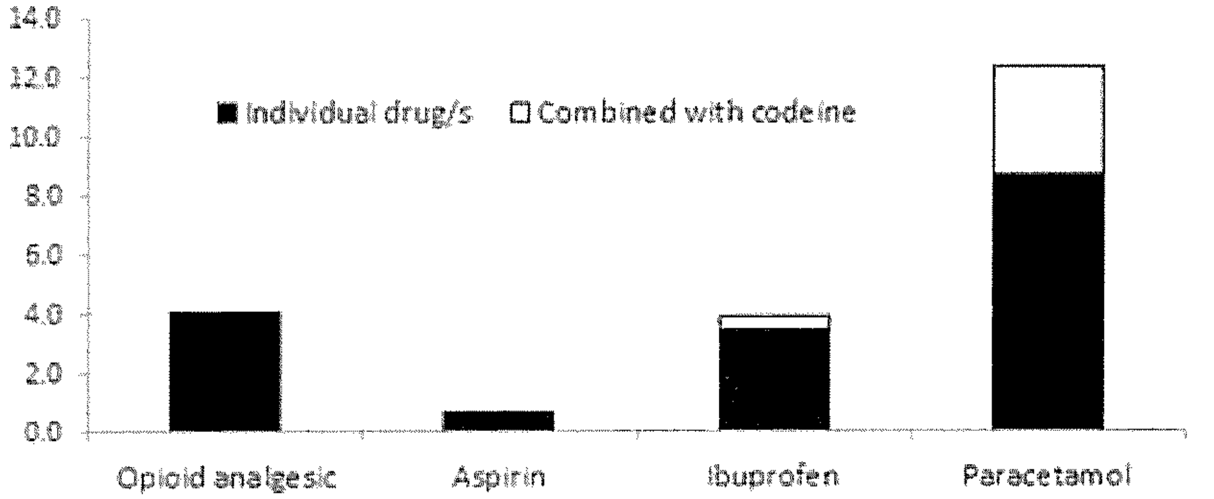

Click here to access/download
Supporting Information
Author Declarations.docx

Related manuscript - sister paper

Click here to access/download
Supporting Information
PONE-D-19-10074_R1 2nd upload.pdf
