# Supplementary material for: National Ambulance Surveillance System: A novel method using coded Australian ambulance clinical records to monitor self-harm and mental health-related morbidity
Source: PLoS One. 2020 Jul 31;15(7):e0236344. doi: 10.1371/journal.pone.0236344 (PMC7394421; doi:10.1371/journal.pone.0236344)
Supplement: Attachment — Submitted filename: Response to Reviewers.docx [file pone.0236344.s004.docx]

We have updated the paragraph on geospatial mapping as suggested by the reviewer. This paragraph now reads:

“The innovative geospatial mapping capability of this surveillance system NASS was enabled by uptake of geo-coordinate collection by many Australian ambulance services. This technology is associated with significant benefits from a public health perspective, but also carries challenges which should be considered and planned for. On the upside, a key strength is that when combined with other coded health and socio-demographic variables, these geo-spatial coordinates allow for an examination of social aetiologies and health disparities between different geographical areas (56). For example, in the context of the present paper, geospatial coordinates may be used to identify local environmental risk factors that are associated with self-harm, for example bridges or rail lines, so that environmental methods can be employed to reduce suicide clusters. An important ethical issue, however, is that geospatial co-ordinates create challenges in managing privacy concerns, given that included subjects may be relatively more identifiable. Consequences of a privacy breach are serious and may include family or friends becoming aware of an individual’s mental health or self-harm issues, loss of employment (e.g. in cases of illicit drug use), or an inability to obtain health insurance (57). As such, NASS data are guided by strict protocols for data de-identification, confidentiality, storage, access and reporting (see Ethics section on page 13).”
